# Prospects of iron solubilizing *Bacillus* species for improving growth and iron in maize (*Zea mays* L.) under axenic conditions

**DOI:** 10.1038/s41598-024-77831-7

**Published:** 2024-11-01

**Authors:** Sammia Ghazanfar, Azhar Hussain, Abubakar Dar, Maqshoof Ahmad, Hammad Anwar, Dunia A. Al Farraj, Muhammad Rizwan, Rashid Iqbal

**Affiliations:** 1 Department of Soil Science, The Islamia University of Bahawalpur, Bahawalpur, 63100 Pakistan; 2 Department of Botany and Microbiology, College of Science, King Saud University, 11451 Riyadh, Saudi Arabia; 3 Institute of Crop Science and Resource Conservation (INRES), University of Bonn, 53115 Bonn, Germany; 4 Department of Agronomy, The Islamia University of Bahawalpur, Bahawalpur, 63100 Pakistan; 5 Department of Life Sciences, Western Caspian University, Baku, Azerbaijan

**Keywords:** Fe solubilization, Biofortification, Siderophores, Exopolysaccharides, P-solubilization, *Bacillus* sp., Plant sciences, Environmental sciences

## Abstract

Iron (Fe) deficiency in calcareous soils is a significant agricultural challenge, affecting crop productivity and nutritional quality. This study aimed to isolate, characterize, and evaluate Fe solubilizing rhizobacterial isolates from maize rhizosphere in calcareous soils as potential biofertilizers. Forty bacterial isolates coded as SG1, SG2, …, SG40 were isolated and screened for siderophore production, with ten showing significant Fe solubilizing capabilities. These isolates were further assessed for phosphate solubilization and exopolysaccharides production. The selected bacterial isolates were also screened under axenic conditions for their ability to improve maize growth. The isolates SG8, SG13, SG24, SG30 and SG33 significantly enhanced growth parameters of maize. Notably, SG30 showed highest increment in shoot length (58%), root length (54%), root fresh and dry biomass (67% and 76%), SPAD value (67%), relative water contents (69%), root surface area (61%), and Fe concentration in shoots (79%) as compared to control. The biochemical characterization of these strains showed that all these strains have capability to solubilize insoluble phosphorus, produce indole-3-acetic acid (IAA), and ammonia with catalase, urease and protease activity. Molecular identification through 16s rRNA gene sequencing confirmed high similarity (99.7–100%) of the selected isolates to various *Bacillus* species, including *B. pyramidoids*, *B. firmicutes*, and *B. cereus*. The study provides a strong base for developing eco-friendly, cost-effective biofertilizers to address Fe deficiency in crops and promote sustainable agriculture.

## Introduction

Iron (Fe) is one of the important micronutrients essential for several physiological and metabolic processes within the plant including respiration, electron transport, photosynthesis and nitrogen fixation^1,2^. Additionally, Fe is involved in antioxidant defense system of plants^3^. According to Riaz et al.^4^ Fe is a vital component of chlorophyll having significant impact on photosynthesis, plant biomass and yield, and enhance human and animal health. The main reason Fe deficiency is its fixation in calcareous high pH soils of Pakistan. Throughout the globe, Fe deficiency is prevalent in human beings and causing hidden hunger or anemia in about 4–5 million people every year with women and children being the most affected^5,6^.

Pakistani soils are deficient in Fe and about 30% of them are calcareous due to high pH and bicarbonates^7^. The Fe availability to plants is influenced by various factors, containing free CaCO_3_, HCO^3−^, and alkaline pH that convert Fe into insoluble hydroxides, which makes it inaccessible to plants^8^. Biofortification is an emerging cost-effective, efficient, and economical method for reducing malnutrition in micronutrients^9,10^. By using beneficial microorganisms, the need for chemical fertilizers is decreased and plant nutrient availability is increased. One method used by microbes and plants to improve Fe acquisition from the soil environment, especially in Fe-limited conditions, is the synthesis of siderophores, which are low-molecular (500–1000 Daltons) Fe chelators that selectively complex Fe (III) with high affinity^11^.

In Fe deficiency-induced chlorosis (IDC), soil microorganisms including bacteria and fungi can produce siderophores, which are comparable to siderophores made by plants but have a high affinity to chelate Fe^12–14^. Generally, fungi produce catecholate siderophores, bacteria exclusively produce siderophores, and plants produce carboxylate siderophores^15,16^. The production of siderophores by bacteria is considered an essential mechanism for maintaining Fe sufficiency. Because siderophores from the catechol group, which are mostly produced by fungi, have a weaker affinity for the element of Fe than do siderophores from the hydroxamate group, which are produced by bacteria, siderophores may be an effective supply of Fe for plants^17^. According to certain study findings, different bacteria, like *Azotobacter*^18,19^, *Pseudomonas*^20^, *Bacillus*^21,22^, *Azospirillum*^23^, *Rhizobium*^24^, *Paenibacillus* can produce siderophores^25^.

Maize is regarded as a vital food crop and a crucial forage, contributing to nearly one-third of global grain production^26,27^. Although the yields per hectare vary greatly, it may be grown in a wide range of agro-ecologies with varying temperatures, elevations, latitudes, land types, and soil types^28^. After rice and wheat, maize ranks as the third most important grain in Pakistan. In 2022–23, maize was cultivated on 1,720 thousand hectares, reflecting a 4.1% increase from the 1,653 thousand hectares used the previous year. Maize production increased by 6.9%, from 9.525 million tons in the previous year to 10.183 million tons. The increase in production is primarily attributed to the expanded cultivation area and improved yield^29^. Millions of people in Africa, South Asia, and Latin America rely on it as a staple food, whereas most of the maize grown in East Asia is used to feed cattle^30^.

To evaluate the ability of Fe biofortification in cereals the most important cereals are wheat and maize because these are staple food of layman in different areas of world. Microbes can fulfill the Fe requirements of plants by the solubilization of insoluble sources of Fe in soil. Keeping in mind the above scenario, the current study was planned to check the effect of Fe solubilizing bacteria for improving plant growth and biofortification of Fe in cereals crops under axenic conditions.

## Materials and methods

### Isolation of Fe solubilizing isolates from maize rhizosphere

Fe solubilizing bacterial isolates were isolated from rhizosphere of maize crop grown in the experimental area of Department of Soil Science, The Islamia University of Bahawalpur, Pakistan, through dilution plate method described by Wollum using nutrient agar media containing insoluble Fe source (FeO)^31^. Agar plates were incubated at 28 ± 2 °C temperature for 24 h in an incubator. Fast growing bacterial colonies with clear zones of Fe solubilization were selected and purified. Purified strains were stored in glycerol at − 30 °C for further experimentation.

### In vitro screening of bacterial isolates against plant growth-promoting attributes

The rhizobacterial isolates were tested for plant growth promoting characteristics on the basis of siderophore production, phosphatase activity and exopolysaccharides production. The siderophore production ability of bacterial isolates was tested both qualitatively and quantitatively. For qualitative siderophore production assay Chromo Azurol Sulfonate (CAS) method described by Schwyn and Neilands was used for all selected rhizobacterial isolates^32^. Further colony and halo zone diameter were recorded and their Fe solubilization efficiency and solubilization index by following the formula described by Sharma et al.^33^. To quantify siderophore activity the CAS broth culture was used^34^. Bacterial isolates grown in nutrient broth for 48 h at 30 ± 2 °C were centrifuged at 3000-rpm for 20 min and 0.5 mL aliquot of supernatant was mix with 0.5 mL of CAS solution and 10 µL of sulfosalicylic acid solution to make final volume. After incubation of 2 h at room temperature, absorbance was calculated at 630 nm using UV-spectrophotometer. A blank was prepared using nutrient broth, CAS and sulfosalicylic acid. Siderophore activity was calculated as

$$Siderophore\;\%=\frac{(Ar-As)}{Ar} \times 100$$

Where, As is the absorbance of sample and Ar is the absorbance of blank.

Phosphate solubilization was determined by the method described by Pikovskaya’s protocol by spot inoculating fresh culture on Pikovskaya’s medium agar plates^35^. The plates were incubated at 28 ± 2 °C for 7 days. Phosphate solubilization was determined by the formation of halo zone around the bacterial colonies. For exopolysaccharides production bacterial isolates were streaked on RCV agar plate media and incubated for 48 h at 28 ± 2 °C. Mucoidal growth around bacterial colonies confirms the exopolysaccharide production^36^.

### Effect of Fe solubilizing bacterial isolates on the growth of maize seedlings under axenic conditions

Under axenic conditions jar trial was conducted to check the effect of Fe solubilizing bacterial isolates on growth of maize. The inoculum of selected rhizobacterial isolates was prepared in LB broth culture and incubated for 48 h at 28 ± 2 °C. Maize seeds were surface disinfected by sodium hypochlorite solution (10%) following Al-Adham et al.^37^ method. The maize seed were inoculated by dipping in respective broth culture for 30 min prior to sowing. Whereas the control seeds were dipped in sterilized LB broth. Jars were filled with 600 g of sand, moisturized by using half-strength solution of Hoagland nutrient solution and autoclaved to remove microbial contamination. Five inoculated seeds of maize were sown in each jar and were placed in a growth room under controlled conditions following completely randomized design. Light and dark periods of 16 and 8 h with day and night temperature (28 and 20 ± 1 °C) and 70% humidity was adjusted. After 26 days plants were harvested and statistically analyzed for growth and physiological attributes. Fe concentration in shoots was also measured by following protocol described by Ryan et al.^38^.

### Characterization of selected bacterial isolates

Selected isolates were evaluated for Auxin production as IAA in the absence and presence of l-tryptophane as proposed by Brick et al.^39^. DF-minimal broth with and without l-tryptophane (0.5%) was prepared separately and inoculated with 48 h old bacterial isolates. The broth was incubated at 28 ± 2 °C for 48 h. After incubation period 3 mL filtrate from culture was taken in a falcon tube and after addition of 2 mL of Salkawoski reagent and left for 30 min to develop color. IAA production was determined by measuring optical density at 530 nm on a spectrophotometer. To test urease activity of bacterial isolates, inoculated in the broth of Christensen’s urea and incubated at 28 ± 2 °C for one day. Urease activity was confirmed by the change in color from the pale orange to pink^40^. The standard protocols were adopted to determine the production of catalase^41^, oxidase^42^, protease^43^, chitinase^44^, ammonia^45^, cellulose^46^, and hydrogen cyanide^47^ in-vitro.

### Molecular identification of isolates

Identification of five best isolates (SG8, SG13, SG24, SG30 and SG33) was caried out using 16S rRNA gene sequencing. DNA from selected bacterial isolates were extracted using proteinase K enzyme method, as described by Chèneby et al.^48^. The 16S rRNA gene was amplified using a polymerase chain reaction using forward and revers set of primers 1492R (TACGGYTACCTTGTTACGACTT) and 27 F (AGAGTTTGATCMTGGCTCAG) as described by Hussain et al.^49^. Purified PCR product was sent to MACROGEN (Seoul, South Korea) for identification, the acquired sequences from bacterial isolates were exposed to a BLAST analysis on the NCBI servers to determine their similarity with its ancestors. Following the procedure described by Roohi et al.^55^ phylogenetic tree was constructed on MEGA-X software using neighbor joining hood method^50^.

### Statistical analysis

The data collected from the study was statistically examined through analysis of variance (ANOVA) under completely randomized design^51^. The significance of treatments was assessed using Tukey’s or honestly significant difference (HSD) test at 5% probability.

## Results

### Isolation of Fe solubilizing bacterial isolates

Forty bacterial isolates coded as SG1, SG2, SG3, SG4, …., SG40 were isolated, purified and screened for their ability to produce siderophore qualitatively.

### In vitro screening of bacterial isolates for plant growth-promoting characteristics

The results from Tables 1 and 2 showed that 10 out of forty bacterial isolates coded as SG4, SG7, SG8, SG13, SG15, SG24, SG30, SG31, SG32 and SG33 showed maximum siderophore production, phosphorus solubilization and exopolysaccharides production. In agar plates maximum bacterial colony diameter (CD) (9.22 mm) was shown by SG33 followed by SG32 and SG24 with 9.02 and 8.85 mm, respectively (Table 1). Maximum halo zone diameter for Fe solubilization (24.5 mm) was shown by isolate SG33 followed by SG32 and SG2 with halo zone diameter of 14.42 to 24.51 mm. Their solubilization efficiency (SE) and solubilization index (SI) were also calculated from halo zone and colony diameter which revealed that maximum value for SE and SI was shown by SG33 that was 265.8% and 3.65, respectively followed by SG4 and SG32 which showed 257.0 and 252.8% SE and 3.57 and 3.52 SI, respectively. While in quantitative essay maximum Fe solubilization (23.55 µg/mL) was observed from isolate SG33 followed by SG32 (22.4 µg/mL) and SG31 (21.2 µg/mL) which are significantly different from each other. Results from Table 2 revealed that all siderophore producing isolates showed the capability to solubilize P from insoluble rock phosphate by forming halo zone on Picovskaya’s media. Maximum halo zone diameter of 21.6 mm was observed from SG33 followed by SG32 and SG30 with 21.1 and 20.7 mm halo zones diameter, respectively. The highest P solubilization index (3.51) was recorded in SG33 followed by SG32 and SG30. Their solubilization efficiency ranged from 197.3 to 251.9% with maximum efficiency shown by SG33 bacterial isolate followed by SG32 and SG7. All Fe solubilizing bacterial isolates except SG13 and SG24 have capability to produce exopolysaccharides and showed positive results for exopolysaccharide production.

**Table 1:** Qualitative and quantitative Fe solubilization efficiency of selected bacterial isolates.

Bacterial isolates	Qualitative essay				Quantitative essay
	Colony diameter (mm)	Halo zone diameter (mm)	Solubilization index	Solubilization efficiency (%)	Fe solubilization (µg/mL)
SG4	5.66 ± 0.012i	14.48 ± 0.136j	3.55 ± 0.020b	255.7 ± 2.090b	12.07 ± 0.128hi
SG7	8.04 ± 0.011e	16.29 ± 0.173g	3.02 ± 0.023i	202.6 ± 2.334i	14.17 ± 0.239f
SG8	6.57 ± 0.012h	14.64 ± 0.207i	3.22 ± 0.035g	222.8 ± 3.558g	12.34 ± 0.105g–i
SG13	6.78 ± 0.036g	16.72 ± 0.198f	3.46 ± 0.021d	246.4 ± 2.166d	12.81 ± 0.131gh
SG15	7.27 ± 0.158f	15.53 ± 0.195h	3.13 ± 0.020h	213.7 ± 2.030h	13.34 ± 0.162fg
SG24	8.90 ± 0.150c	21.65 ± 0.176c	3.43 ± 0.048e	243.2 ± 4.898e	19.29 ± 0.194d
SG30	7.29 ± 0.086f	17.24 ± 0.024e	3.36 ± 0.024f	236.6 ± 2.495f	15.54 ± 0.108e
SG31	8.55 ± 0.064d	20.04 ± 0.008d	3.34 ± 0.017f	234.4 ± 1.723f	21.15 ± 0.401c
SG32	9.06 ± 0.011b	22.86 ± 0.094b	3.52 ± 0.013c	252.4 ± 1.358c	22.43 ± 0.225b
SG33	9.26 ± 0.005a	24.55 ± 0.132a	3.65 ± 0.014a	265.0 ± 1.362a	23.55 ± 0.256a
HSD (*p* ≤ 0.05)	**0.147**	**0.158**	**0.031**	**3.131**	**1.058**

Means with similar letters are statistically non-significant to each other (*p* < 0.05).

**Table 2:** Phosphorus solubilization and exopolysaccharides production efficiency of selected bacterial isolates.

Bacterial isolates	Colony diameter (mm)	Halo zone diameter (mm)	Solubilization index	Solubilization efficiency (%)	Exopolysaccharides (EPS)
SG4	6.36 ± 0.017i	12.44 ± 0.171j	2.95 ± 0.031f	195.60 ± 3.173g	+
SG7	6.28 ± 0.035i	14.73 ± 0.136h	3.34 ± 0.035b	234.67 ± 3.502c	+
SG8	7.06 ± 0.011h	14.26 ± 0.008i	3.02 ± 0.003e	201.89 ± 0.389f	+
SG13	7.27 ± 0.026g	16.66 ± 0.171g	3.29 ± 0.031c	228.96 ± 3.183d	−
SG15	7.69 ± 0.012f	17.37 ± 0.185f	3.25 ± 0.023cd	225.73 ± 2.399de	+
SG24	8.30 ± 0.015e	18.60 ± 0.096e	3.24 ± 0.014d	224.09 ± 1.460e	−
SG30	9.14 ± 0.015b	20.76 ± 0.135c	3.27 ± 0.011cd	227.12 ± 1.134de	+
SG31	9.51 ± 0.020a	19.38 ± 0.083d	3.03 ± 0.012e	203.64 ± 1.248f	+
SG32	8.85 ± 0.018c	21.13 ± 0.008b	3.38 ± 0.004b	238.83 ± 0.414b	+
SG33	8.60 ± 0.023d	21.64 ± 0.034a	3.51 ± 0.003a	251.43 ± 0.279a	+
HSD (*p* ≤ 0.05)	**0.157**	**0.135**	**0.040**	**3.873**	−

Means with similar letters are statistically non-significant to each other (*p* < 0.05).

### Effect of Fe solubilizing bacterial isolates on growth of maize under axenic conditions

The effectiveness of Fe solubilizing bacterial isolates was further evaluated for their ability to increase maize growth under controlled conditions. Results from Table 3 showed that inoculation of Fe-solubilizing rhizobacterial isolates showed significant improvement in maize growth. Statistical analysis showed that maximum shoot length was observed from inoculation with SG30 isolate which showed 58% increase followed by SG33 which showed 57% increase in shoot length over control. Minimum improvement was seen in SG4 with only 15% increase in shoot length. Similarly, all tested isolates showed notable enhancement in root length ranging from 8 to 60%. The highest increase in root length (61%) was observed from inoculation of SG30 followed by 54 and 50% increase shown by SG33 and SG8, respectively, as compared to control. Bacterial isolate SG24 and SG31 showed 38 and 25% increase in root length of maize crop, respectively.

**Table 3:** Effect of Fe solubilizing bacterial isolates on growth attributes of maize crop under axenic conditions.

Treatments	Shoot length (cm)	Root length (cm)	Shoot fresh weight (g)	Root fresh weight (g)	Shoot dry weight (g)	Root dry weight (g)
Control	19.63 ± 0.318e	8.29 ± 0.187g	0.488 ± 0.083e	0.380 ± 0.015g	0.365 ± 0.008g	0.283 ± 0.008g
SG4	22.55 ± 0.294de	9.42 ± 0.198f	0.52 ± 0.006de	0.457 ± 0.016c–e	0.41 ± 0.006fg	0.297 ± 0.009g
SG7	24.05 ± 0.336d	8.97 ± 0.042 fg	0.51 ± 0.006e	0.483 ± 0.014cd	0.39 ± 0.017g	0.317 ± 0.009fg
SG8	29.02 ± 0.303b	12.47 ± 0.088b	0.68 ± 0.012b	0.507 ± 0.009c	0.50 ± 0.003de	0.383 ± 0.008de
SG13	28.55 ± 0.400b	10.89 ± 0.177cd	0.62 ± 0.006c	0.562 ± 0.009b	0.60 ± 0.008ab	0.403 ± 0.003cd
SG15	23.13 ± 0.188d	9.23 ± 0.170f	0.58 ± 0.015cd	0.430 ± 0.01e–g	0.48 ± 0.005de	0.364 ± 0.007d–f
SG24	28.67 ± 0.387b	11.44 ± 0.292c	0.72 ± 0.009b	0.583 ± 0.009ab	0.52 ± 0.006cd	0.477 ± 0.008ab
SG30	31.01 ± 0.199a	13.31 ± 0.158a	0.79 ± 0.088a	0.634 ± 0.006a	0.58 ± 0.012bc	0.500 ± 0.023a
SG31	26.75 ± 0.415c	10.33 ± 0.110de	0.57 ± 0.088c–e	0.442 ± 0.006d–f	0.45 ± 0.011ef	0.330 ± 0.011e–g
SG32	23.25 ± 0.179d	9.71 ± 0.092ef	0.55 ± 0.007de	0.400 ± 0.011fg	0.42 ± 0.012fg	0.337 ± 0.008e–g
SG33	30.83 ± 0.167a	12.81 ± 0.135ab	0.83 ± 0.015a	0.603 ± 0.003ab	0.65 ± 0.019a	0.443 ± 0.009bc
HSD (*p* ≤ 0.05)	**1.530**	**0.824**	**0.058**	**0.050**	**0.056**	**0.054**

Means with similar letters are statistically non-significant to each other (*p* < 0.05).

Maximum shoot fresh weight was observed from inoculation with SG33 which showed 69% increase as compared to control followed by SG30, SG24 and SG8 with 61, 48 and 40% increase, respectively. While lowest improvement was shown by SG7 with only 4% increase as compared to control. Shoot dry weight was also significantly improved by the inoculation with Fe solubilizing bacterial isolates with maximum increase of 77% observed from SG33 followed by SG13 and SG30 which showed 65 and 58% improvement respectively over control. Inoculation of SG30 bacterial isolate showed maximum root fresh and dry weight with 67 and 76% increase, respectively, as compared to control.

### Effect of Fe solubilizing bacterial isolates on physiological attributes and Fe concentration in shoots and roots of maize under axenic conditions

**Fig. 1:**
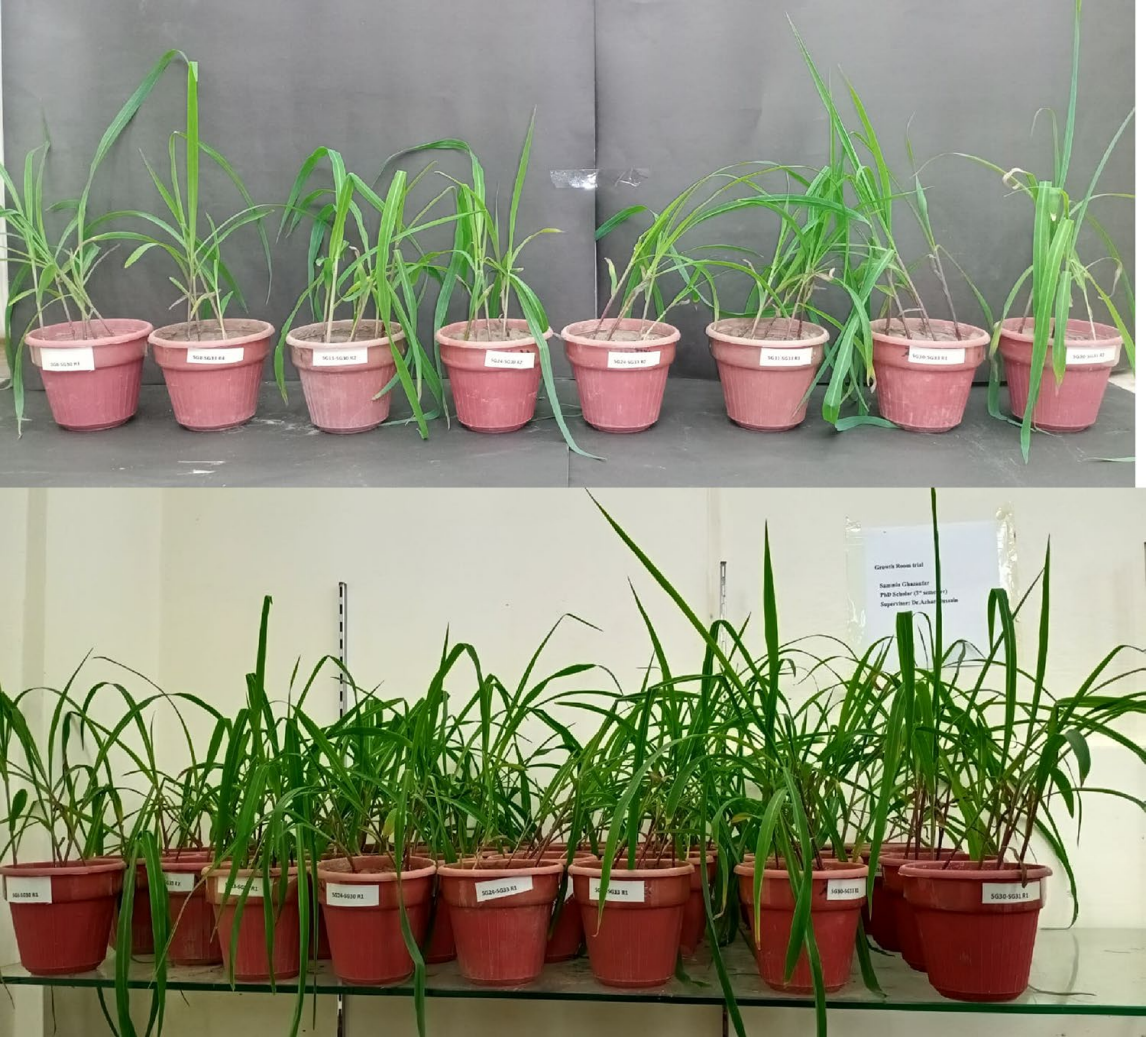
Effect of different Fe solubilizing isolates on growth of maize seedlings under axenic conditions.

Data regarding physiological attributes revealed that all the inoculated bacterial isolates significantly improved leaf area, membrane stability index, root surface area and root volume of maize crop (Table 4.) and SPAD value, relative water contents and Fe concentration in shoots and roots of maize crop (Fig. 1). Fe solubilizing bacterial isolate SG33 showed a maximum increase in leaf area that was 66% followed by SG24 with 65% increase in leaf area over control. While SG13 and SG30 showed 58% improvement in leaf area over control. Membrane stability index was also significantly improved by the inoculation of Fe solubilizing bacterial isolates with a maximum of 60% increase from SG33 followed by SG24, SG30 and SG8 with 49, 36 and 33% increase, respectively.

Inoculation of SG30 bacterial isolate showed maximum increase in root surface area that was 61% followed by SG33, SG24 and SG8 with 60, 54 and 49% increase, respectively, as compared to control. Similarly root volume was also significantly improved by inoculation of Fe solubilizing bacterial isolates with a maximum increase of 57% observed from SG33 over control followed by SG30 and SG24 which showed 54 and 49% increase respectively.

**Table 4:** Effect of Fe solubilizing bacterial isolates on physiological attributes of maize crop under axenic conditions.

Treatments	Leaf area (cm^2^)	Membrane stability index	Root surface area (cm^2^)	Root volume (cm^2^)
Control	160 ± 1.452f	41.07 ± 0.383g	97 ± 3.605g	10.54 ± 0.326f
SG4	192 ± 1.691de	49.33 ± 0.210de	120 ± 0.967f	12.29 ± 0.127e
SG7	188 ± 1.660e	47.33 ± 0.380d–f	127 ± 1.581ef	13.03 ± 0.120e
SG8	245 ± 1.603bc	54.50 ± 0.418bc	145 ± 1.997b–d	15.62 ± 0.098bc
SG13	253 ± 3.068ab	51.00 ± 0.182cd	140 ± 1.041cd	14.95 ± 0.078cd
SG15	201 ± 2.027d	44.00 ± 0.316fg	129 ± 1.626ef	12.33 ± 0.120e
SG24	265 ± 4.041a	59.00 ± 0.182b	150 ± 2.819a–c	15.81 ± 0.103a–c
SG30	254 ± 2.081ab	56.00 ± 0.365b	156 ± 1.560a	16.21 ± 0.142ab
SG31	233 ± 2.231c	46.33 ± 0.210d–f	136 ± 1.514de	14.40 ± 0.153d
SG32	191 ± 2.603de	45.00 ± 0.182e–g	127 ± 1.495ef	15.63 ± 0.088bc
SG33	266 ± 3.754a	65.63 ± 0.231a	152 ± 1.553ab	16.60 ± 0.292a
HSD (*p* ≤ 0.05)	**13.160**	**4.678**	**9.816**	**0.856**

Means with similar letters are statistically non-significant to each other (*p* < 0.05).

**Fig. 2:**
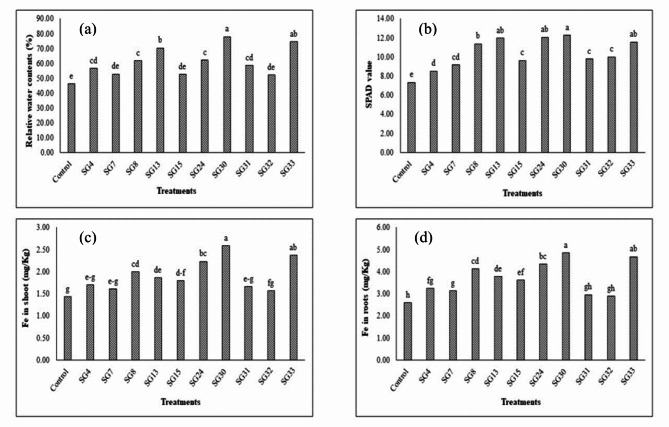
Effect of Fe solubilizing bacterial isolates on relative water contents (**a**), SPAD value (**b**), Fe in shoots (**c**) and Fe in roots (**d**) of maize crop under axenic conditions (*p* < 0.05).

Data from Fig. 2 showed that the highest SPAD value was observed from inoculation of SG30 with 67% increase as compared to control followed by SG24, SG13 and SG33 which showed 64, 62 and 58% increase, respectively. Bacterial isolate SG30 showed highest value for relative water contents with 69% increase over control. Further best results were attained from inoculation of SG33 with 62% increase as compared to control. The lowest value for relative water contents was observed from inoculation of SG32 with 13% increase. Significant increase in Fe concentration in plant shoots and roots was observed from inoculated treatments. Maximum increase in Fe concentration in shoots and roots was observed from inoculation of SG30 isolate which showed 79 and 86% increase in Fe uptake as compared to control. Next better results were observed from SG33 and SG24 with 65 and 55% increase respectively from shoots while 80 and 67% respectively from roots. While minimum improvement was observed by SG32 with 8% and 11% increase from shoots and roots respectively.

### Characterization of selected bacterial isolates

From the results of jar trail five best performing bacterial isolates coded as SG8, SG13, SG24, SG30 and SG33 were selected and further evaluated for in-vitro plant growth promoting characters. The results from Table 5 showed that all tested bacterial isolates showed positive results for IAA production in presence and absence of l-tryptophane. In presence of l-tryptophan highest IAA was produced from SG33 isolate that was 21.77 µg mL^−1^ followed by SG30 and SG8 which showed 20.17 and 17.31 µg mL^−1^ respectively. Without l-tryptophan a maximum of 18.96 µg mL^−1^ of IAA was produced from SG33 followed by SG30 with 18.67 µg mL^−1^ of IAA while SG13 showed minimum IAA production (12.05 µg mL^−1^). All tested Fe solubilizing bacterial isolates showed positive results for catalase, urease and protease activity. Out of five selected isolates SG13 showed negative results for oxidase activity while all remaining showed positive results. Hydrogen cyanide and ammonia production was observed positive from all tested isolates while for cellulose production only SG8 showed negative results. Chitinase activity was also observed positive from all tested Fe solubilizing bacterial isolates except SG8 which showed negative results for chitinase activity.

**Table 5:** In vitro plant growth-promoting attributes of selected Fe-solubilizing bacterial isolates.

Characters		SG8	SG13	SG24	SG30	SG33
Indole-3-acetic acid production (µg/mL)						
With l-tryptophane		17.31 ± 0.420ab	13.72 ± 0.433c	14.45 ± 0.346bc	20.17 ± 0.273b	21.77 ± 0.494a
Without l-tryptophane		16.00 ± 0.129b	12.05 ± 0.197c	12.71 ± 0.49c	18.67 ± 0.458a	18.96 ± 0.352a
Catalase activity		+	+	+	+	+
Urease activity		+	+	+	+	+
Protease activity		+	+	+	+	+
Oxidase activity		+	−	+	+	+
Hydrogen cyanide production		+	+	+	+	+
Ammonia production		+	+	+	+	+
Chitinase activity		−	+	+	+	+
Cellulose activity		−	+	+	+	+

(+) indicates production while (−) indicates no production.

### Identification of selected bacterial isolates

**Fig. 3:**
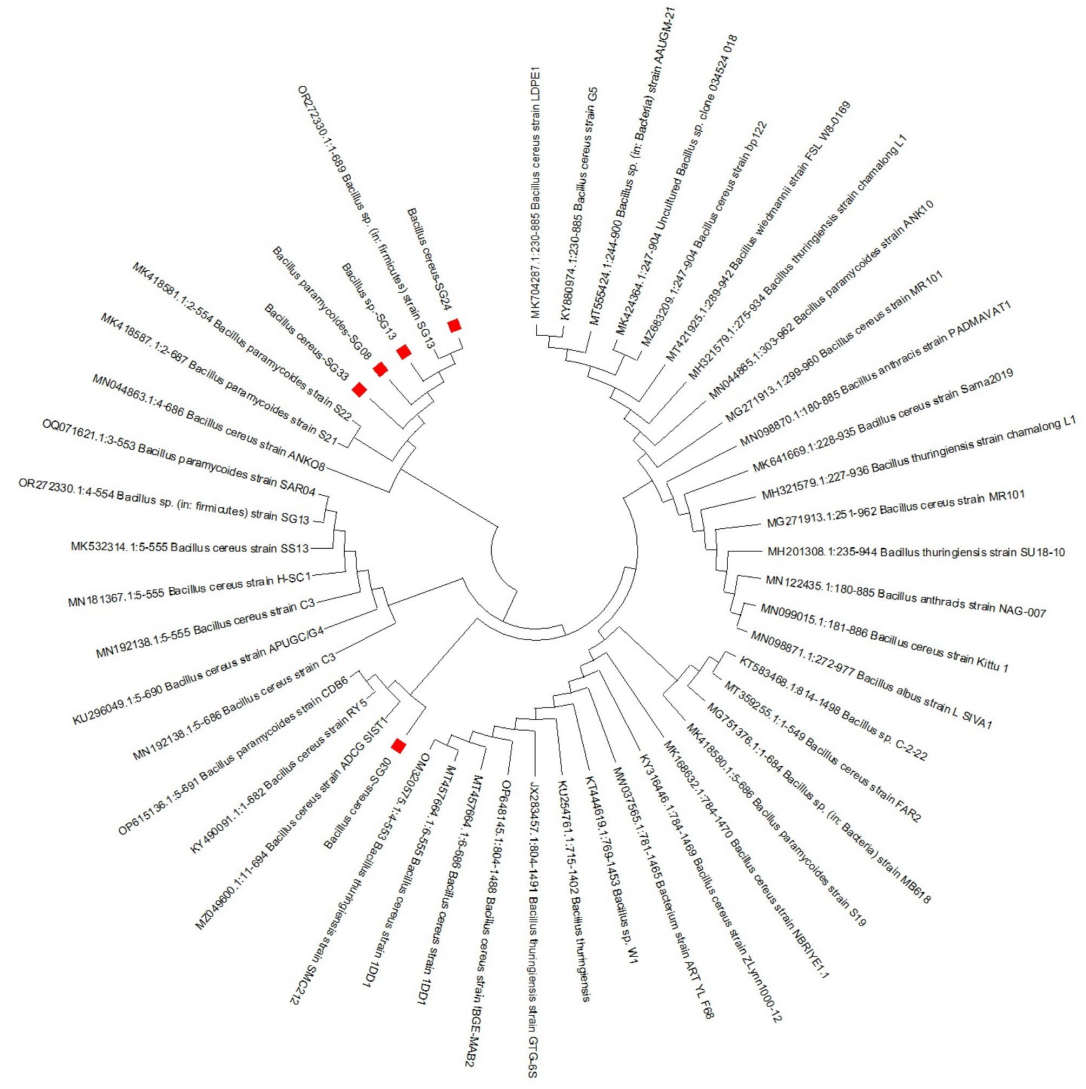
Neighbor-joining phylogenetic analysis was conducted using the multiple alignment of 16S rRNA gene sequences from *Bacillus* spp. and other bacterial strains obtained from the Gene Bank database.

Five highly promising Fe solubilizing bacterial isolates, were identified as gram-positive. The isolate SG8 exhibited a 99.85% resemblance to *Bacillus pyramidoids* and was stored in GenBank with the accession number OR272329. While SG13 was identified as *Bacillus firmicutes* with 100% similarity and was assigned the accession number OR272330. Isolate SG24 showed 99.85%, SG30 showed 100%, and SG33 showed 99.72% similarity with *Bacillus cereus*. The isolates were submitted to GenBank with the accession numbers OR272331, OR272332, and OR272333, respectively (Fig. 3). The phylogenetic tree generated from the BLAST investigation revealed that these isolates are part of established and previously recognized bacterial species.

## Discussion

Fe can only be absorbed by plants in its ferrous form (Fe^2+^). Thus, alterations in the pH of rhizosphere soil led to its conversion in ferric iron (Fe^3+^) into soluble iron (Fe^2+^). The pH of the rhizosphere soil is influenced by the organic acids released by microbes. Soil microorganisms play an important role in solubilizing Fe, thereby improving growth and yield of plant^52^. This study highlights the Fe solubilization capabilities of *Bacillus* species, *Bacillus pyramidoids*, *Bacillus firmicutes*, and *Bacillus cereus* obtained from the rhizosphere of maize crop. This work’s primary goal was to identify and isolate bacterial strains from calcareous soils that have a high potential for producing siderophores. These strains could be utilized as an environmentally friendly substitute for synthetic ligands as Fe biofertilizer. In addition to its Fe complexing ability, the traits that support plant growth were examined. In the current study, forty strains from the rhizosphere of maize were isolated, based on varying colony morphologies. The isolated rhizobacteria underwent screening for their capacity to solubilize Fe qualitatively. On the selected bacteria, the CAS test and the comparison in halo zone formation were carried out. Ten bacterial isolates were chosen from those that qualitatively produced a greater halo than the control: SG4, SG8, SG13, SG24, SG30, SG31, SG32, and SG33 (Table 1). The qualitative and quantitative screening for Fe solubilization using CAS assay proved to be an effective method for initial selection of potential Fe solubilizing isolates.

The development of orange-colored halo zones around bacterial colonies on CAS agar media aligns with the observations reported by Sarwar et al.^53^ indicating the production of siderophores. The varying halo sizes observed among our isolates suggested the differences in siderophore producing efficiency, with larger halos potentially indicating higher siderophore production. Research findings of Priyanka and Nakkeeran^54^ and Masmoudi et al.^55^ confirmed that *Ochrobactrum* sp. and *Bacillus* sp. have the capability to synthesis the higher amount of siderophores.The study of Rajkumar et al.^56^ that rhizobacteria produce various types of siderophores including salicylate carboxylate and hydroxamate. This variability in siderophore production among rhizobacterial isolates has also been previously noted by Jabborova et al.^57^ who reported that number of rhizospheric bacteria synthesis secondary metabolites called as siderophores, which act like ferric-iron chelating agents and produce at low Fe stress and is crucial for enhancing Fe bioavailability in the rhizosphere^58^. Our in-vitro studies revealed significant variability in growth-promoting characteristics among the tested Fe solubilizing isolates. The ability of isolates coded as SG33, SG31, SG24, SG13, and SG8 to solubilize phosphate is particularly noteworthy, as it suggests that these strains could address multiple nutrient deficiencies simultaneously. These findings are in line with work of Pahari and Mishra^59^ and Afzal et al.^60^ who also reported on the dual ability of certain rhizobacteria to solubilize both Fe and phosphorus. The production of indole-3-acetic acid by selected bacterial strains, both in the absence and presence of L-tryptophane (Table 5), demonstrates their potential to influence plant growth through phytohormone production. The greater production of IAA in the presence of l-tryptophane aligns with the findings of Javed et al.^5^ highlighting the role of this amino acid (l-tryptophan) as a precursor for auxin biosynthesis in rhizobacteria. This auxin production capability, combined with other traits such as protease, chitinase and catalase activity as well as ammonia and hydrogen cyanide production, underscores the multifaceted growth-promoting potential of these isolates. Research described by Pahari and Mishra^59^ and Afzal et al.^60^ had shown that PGPR can improve development of plant both directly and indirectly. This enhancement occurs through mechanisms such as nutrient solubilization, phytohormone production, and enzyme production. The results of this research showed that inoculation of siderophore producing bacteria significantly increase growth attributes of maize crop including root and shoot length (58 and 54%) their biomass (76%), root surface area (61%) and leaf area. Similar results have also been founded in previous work of Kamboh et al.^61^, Sharma et al.^62^ and Rana et al.^63^. Similar findings were also reported by Kusale et al.^64^ who found that sesame plants treated with siderophore-producing microorganisms showed significantly longer shoots and roots and biomass in addition to higher SPAD values. Our findings are consistent with earlier studies by Saleem et al.^65^ and Mushtaq^66^ who found that siderophore enhanced plants’ absorption of Fe, leading to an increase in chlorophyll levels, leaf area, and photosynthetic rate.

The current study suggests that an increase in physiological attributes including relative water content and membrane stability index could be attributed to enhanced phosphorus solubilization, uptake, and translocation of Fe, as well as the production of auxin and other phytohormones^67–69^. The Fe solubilizing bacterial isolates significantly increased the availability of Fe and its concentration in shoots and roots of maize crop (79 and 86%). In soil majority of Fe exists in Fe^3+^ oxidation state which is not readily available to plants even though it is essential for numerous physiological processes within the plant body^70^. Bacteria commonly acquire Fe by the production of siderophores low molecular weight compounds with a higher affinity for complexing Fe^3+^^71^. In siderophore producing bacteria, the Fe^3+^ in the Fe^3+^ siderophore complex, bound to the bacterial-membrane, is minimized to Fe^2+^. This Fe^2+^ is then released into the cell via a gating mechanism that coordinates the inner and outer membranes. Thus, siderophores function as extracellular solubilizing agent, mobilizing minerals Fe under Fe-deficient environment and subsequently enhancing plant growth.

## Conclusion

This study demonstrates the significant potential of Fe-solubilizing rhizobacteria isolated from maize rhizosphere in calcareous soils as effective biofertilizers. The selected isolates, particularly SG33, SG30 and SG24 exhibited remarkable capabilities in siderophore production, phosphorus solubilization and other plant growth promoting traits. These strains significantly enhanced maize growth and physiological attributes under axenic conditions, likely due to improved Fe availability and uptake. The multifaceted plant growth promoting characteristics of these isolates including IAA production, catalase activity and ammonia production, further underscores their potential as bioinoculants. These findings pave the way for developing eco-friendly and cost-effective biofertilizers that can mitigate Fe deficiency in crops and promote sustainable agriculture. Further field studies are required to validate the efficacy of these promising isolates under diverse environmental conditions and to explore their potential in enhancing crop productivity and nutritional quality.

## Data Availability

All the data related to this work can be sourced from the corresponding authors. The datasets generated and/or analyzed during the current study are available in the NCBI repository (https://www.ncbi.nlm.nih.gov/) with accession numbers [OR272329, OR272330, OR272331, OR272332, OR272333 with persistent web link; https://www.ncbi.nlm.nih.gov/nuccore/OR272329.1/; https://www.ncbi.nlm.nih.gov/nuccore/OR272330.1/; https://www.ncbi.nlm.nih.gov/nuccore/OR272331.1/; https://www.ncbi.nlm.nih.gov/nuccore/OR272332.1/; https://www.ncbi.nlm.nih.gov/nuccore/OR272333.1/; respectively].
